# Estimating the basic reproduction rate of HFMD using the time series SIR model in Guangdong, China

**DOI:** 10.1371/journal.pone.0179623

**Published:** 2017-07-10

**Authors:** Zhicheng Du, Wangjian Zhang, Dingmei Zhang, Shicheng Yu, Yuantao Hao

**Affiliations:** 1 Department of Medical Statistics and Epidemiology & Health Information Research Center & Guangdong Key Laboratory of Medicine, School of Public Health, Sun Yat-sen University, Guangzhou, Guangdong Province, China; 2 Key Laboratory of Tropical Diseases and Control of the Ministry of Education, Guangzhou, China; 3 Public Health Surveillance and Information Services Center, Chinese Center for Disease Control and Prevention, Beijing, China; Centers for Disease Control and Prevention, UNITED STATES

## Abstract

Hand, foot, and mouth disease (HFMD) has caused a substantial burden of disease in China, especially in Guangdong Province. Based on notifiable cases, we use the time series Susceptible-Infected-Recovered model to estimate the basic reproduction rate (R0) and the herd immunity threshold, understanding the transmission and persistence of HFMD more completely for efficient intervention in this province. The standardized difference between the reported and fitted time series of HFMD was 0.009 (<0.2). The median basic reproduction rate of total, enterovirus 71, and coxsackievirus 16 cases in Guangdong were 4.621 (IQR: 3.907–5.823), 3.023 (IQR: 2.289–4.292) and 7.767 (IQR: 6.903–10.353), respectively. The heatmap of R0 showed semiannual peaks of activity, including a major peak in spring and early summer (about the 12^th^ week) followed by a smaller peak in autumn (about the 36^th^ week). The county-level model showed that Longchuan (R0 = 33), Gaozhou (R0 = 24), Huazhou (R0 = 23) and Qingxin (R0 = 19) counties have higher basic reproduction rate than other counties in the province. The epidemic of HFMD in Guangdong Province is still grim, and strategies like the World Health Organization’s expanded program on immunization need to be implemented. An elimination of HFMD in Guangdong might need a Herd Immunity Threshold of 78%.

## Introduction

Hand, foot and mouth disease (HFMD) is a major public health issue in China, affecting over two million children annually [1, 2]. Particularly, the incidence of HFMD in Guangdong Province exceeded 30/10,000 per year, which was more than three times the national average [3, 4]. An efficient intervention, a necessary and important action to prevent and control the spread of diseases, hinges on a complete understanding of the transmission and persistence of HFMD.

Transmission of HFMD cases occurs by direct contact with the mucus, saliva, or feces of an infected individual, or through indirect contact via contaminated surfaces. The basic reproduction rate (R0) is used to measure the transmission potential of a disease. R0 is the number of expected secondary infections resulting from a single infectious case [5]. In general, if R0 is greater than one, the disease will continue to spread, and if R0 is less than one, the disease will eventually disappear. Also, R0 can be used to estimate the herd immunity threshold (HIT) needed to stop transmission of communicable diseases [6]. Both R0 and HIT are the key measures used in infectious disease control, immunization and eradication.

Dynamic modelling of infectious diseases has contributed greatly to estimating R0 [7]. Several types of mathematical models have become valuable aid to understanding and fighting transmission of HFMD: A Susceptible-Infected-Recovered (SIR) epidemic model to predict the number of infected in Sarawak, Malaysia, was proposed by Tiing et al. [8]. Roy et al. established a Susceptible-Exposed-Infectious-Recovered (SEIR) model to understand the dynamics of HFMD among young children in Khulna, Bangladesh [9]. In addition, Yang et al. established a Susceptible-Exposed-Infectious-Quarantined-Recovered (SEIQR) model and estimated the R0 for the HFMD transmission in mainland China [10].

However, the transmission dynamics varied across geolocation due to different socio-economic situations, demography, health resources, and people's lifestyles. Targeted strategies for prevention and control would benefit from understanding the transmission dynamics for different geographical levels (i.e. province, city, and county). It is necessary to study the transmission dynamics of HFMD in Guangdong due to its serious disease burden. We used a time series Susceptible-Infected-Recovered model (TSIR) to estimate the R0 and HIT of HFMD in Guangdong based on varying province, city, and county geographical levels. The TSIR was developed by including the time series as a covariate in a nonparametric autoregressive modelling approach to improve forecasting performance, and can be used to estimate transmission dynamics [11]. Moreover, the TSIR model is easier to understand because it is based on simple linear regression [12].

To our knowledge, these principal epidemiological parameters (R0 and HIT) of HFMD in Guangdong Province are yet to be addressed and have not been estimated. In this paper, we adopted the TSIR model to study the transmission dynamics of HFMD in Guangdong. We estimated the spatial pattern of transmission dynamics based on different geographical scales including province, city, and county levels.

## Materials and methods

### Ethics statement

This study was based on official HFMD surveillance data in Guangdong, China. Analyses were conducted at aggregate level and no confidential information was involved. The research study protocol was approved by the Institutional Review Board of the School of Public Health, Sun Yat-sen University. All methods were performed in accordance with the relevant ethical guidelines and regulations.

### Study site

Guangdong Province, situated at latitude 20° 15’ 0” to 25° 51’ 0” N and longitude 109° 75’ 0” to 117° 33’ 0” E, has a population of 104 million (from 2010 census data). According to the Annual Statistical Report of Guangdong (http://www.gdstats.gov.cn/tjsj/gdtjnj/), the province can be generally divided into four parts: the Pearl River Delta Region, Eastern Region, Western Region, and Mountainous Region. These four regions have different socio-economic profiles, and the Pearl River Delta Region, located around the center of the province, bears a much higher HFMD burden than that borne by the other three areas [13], the accumulative incidence from 2009 to 2012 being 39/10,000 in the Pearl River Delta Region; 7/10,000 in the Eastern Region; 12/10,000 in the Western Region; and 14/10,000 in the Mountainous Region. The population in Guangdong was non-vaccinated from 2009 to 2012 [14].

### Data collection

Case-based HFMD surveillance data from 2009 to 2012 were obtained from the National Center for Public Health Surveillance and Information Services and the China Center for Disease Control and Prevention (China CDC) (S1 Dataset). This enhanced national surveillance system has been described in detail elsewhere [15]. Population (*pop*) and birth statistics were obtained from the Annual Statistical Report of Guangdong and the National Population and Health Science Data Sharing Platform (S1 Dataset) [16]. The *cases*_*t*_ was adjusted to the infected part *I*_*adj*,*t*_ for the TSIR model by multiplying by a derivative function:

$$\rho \text{=}\frac{d{\hat{Y}}_{cumBirths}}{dX_{cumCases}}$$ (1)

$$I_{\text{adj},t}\text{=}cases_{t}\times \rho +1$$ (2)

We calculated the cumulate number of cases (*X*_*cumCases*_) and births (*Y*_*cumBiths*_) with the numbers of HFMD cases (*cases*_*t*_) and the births. The ${\hat{Y}}_{cumBirths}$ was obtained by fitting the locally weighted scatterplot smoothing (LOWESS) non-parametric regression:

$${\hat{Y}}_{cumBirths}\text{=}Lowess\left(X_{cumCases},Y_{cumBirths},f=2/3\right)$$ (3)

The susceptible part (*S*) for the TSIR model was calculated by multiplying *pop* and a proportion (*p*) which was simulated from 1% to 40% in our study (*S* = *p* × *pop*). To get a visible spatial pattern of the transmission dynamics of HFMD in Guangdong, we acquired the county-level shapefile map of Guangdong Province from OpenStreetMap (OpenStreetMap Foundation, London, United Kingdom).

### Time series susceptible-infected-recovered model

The TSIR model is a discrete-time version of the continuous-time SIR model in which individuals are born and enter the susceptible class of individuals, become infected and infectious with a disease, and recover and are removed thereafter [12]. For HFMD, the characteristic time scale of the disease (i.e. the duration of the transition from infection to recovery and temporary immunity) is about two weeks [17]. Therefore, any new infection must arise from an interaction between a susceptible and an infected individual sometime within the previous bi-week. So, we aggregated the data into bi-weekly time steps, and there are twenty-six period (*period*_1,…,26_) per year, then the future number of infected can be explained as a function of the previous number of infected. The function contained three components including the seasonal transmission (*β*_1_×*period*_1_+…+*β*_26_×*period*_26_), the non-seasonal transmission (*α*×*I*_*adj*,*t-1*_) and the priori known component (*S* + *Z*). The appropriate discrete dynamic two-dimensional compartment model for a childhood disease is thus given by:

$$\text{log}\left(I_{\text{adj},t}\right)=\beta_{1}\times period_{1}+\ldots +\beta_{26}\times period_{26}+\alpha \times \text{log}\left(I_{\text{adj},t-1}\right)+\text{offset}\left(\text{log}\left(S+Z\right)\right)$$ (4)

Here the *Z* was calculated by ${\hat{Y}}_{cumBirths}-Y_{cumBirths}$, indicating the newborn population. We were interested in estimating the seasonal transmission parameters *β*_1,…,26_ and the mixing parameters rate *α* which is a correction parameter accounting for non-seasonal heterogeneities [11, 18]. The estimated values of *α* and *β*_1,…,26_ obtained from each model were available in S1 Table.

### Basic reproduction rate and herd immunity threshold

R0 is used to measure the transmission potential of a disease, and thought of as the number of secondary infections produced by a typical case of an infection in a population that is totally susceptible [19]. It can therefore be measured by counting the number of secondary cases following the introduction of an infection into a totally susceptible population. For those mathematical models with differential equations (i.e. Li et al. [20, 21] and Wu et al. [22]), the R0 was derived according to the concepts of the next generation matrix. While the next generation matrix was replaced by the seasonal transmission parameters *β*_1,…,26_ in our study. The *β*_1,…,26_ were the regression coefficients of the Eq 4, and could be estimated by using the algorithm of iteratively reweighted least squares (IRLS) [23]. Thus, the R0 in our study is given by:

$$R0_{1,\ldots ,26}=\beta_{1,\ldots ,26}\times \bar{S}$$ (5)

For each model (i.e. serotype-, city-, and county- specific models), we calculated the median R0 of R0_1,…,26_ as the final result.

HIT is the proportion of a population that must be vaccinated to become immune so that an infectious disease can become stable in that community [6]. When the proportion is reached by vaccination, each case leads to a single new case and the infection becomes stable. We calculated the HIT by:

$$HIT=1-\frac{1}{R0}$$ (6)

### Software and packages

All of the statistical analyses were conducted in R version 3.3.2 (R Core Team, Vienna, Austria), using packages including base, tsiR, rgeos, maptools, RColorBrewer, and stddiff.

## Results

### The goodness of fit of the TSIR model for HFMD in Guangdong

**Fig 1:**
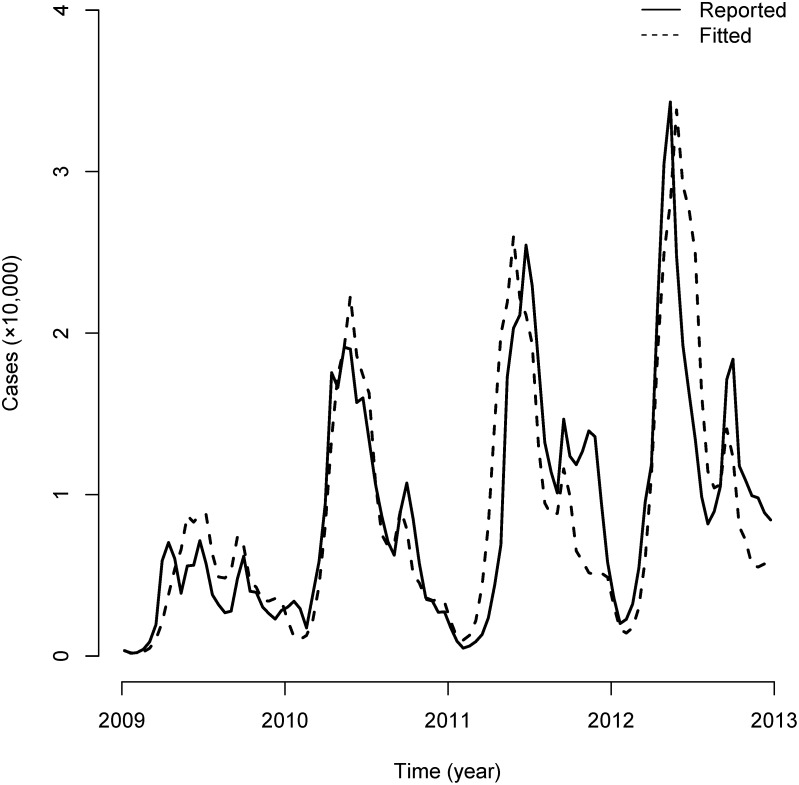
TSIR model fit for Guangdong Province. The solid line represents bi-weekly reported HFMD cases, and the dash line represents bi-weekly fitted cases from the TSIR model.

The bi-weekly HFMD cases fitted by the TSIR model were matched to the reported cases in Guangdong Province (Fig 1). We calculated the standardized difference (<0.2 represents the balance between reported and fitted groups) for the overall duration (0.009). Other fitting indicators, including mean absolute percentage error (MAPE, 40.94%) and Pearson's product-moment correlation (r = 0.858, P<0.001), were also calculated.

### The serotype-specific R0 for HFMD in Guangdong

The median R0 of total cases in Guangdong was 4.621 (Interquartile Range, IQR: 3.907–5.823). The serotype-specific median R0 of enterovirus 71 (EV71), coxsackievirus A16 (CVA16), and other serotypes in Guangdong were 3.023 (IQR: 2.289–4.292), 7.767 (IQR: 6.903–10.353) and 7.368 (IQR: 5.988–8.369). (Table 1)

**Table 1:** Serotype-specific R0 and HIT of HFMD in Guangdong.

Group	R0					HIT (%)
	Minimum	Q_25_*	Median	Q_75_*	Maximum	
Total (N = 924,804)	2.072	3.907	4.621	5.823	7.551	78
EV71 (N = 8,217)	1.285	2.289	3.023	4.292	6.558	67
CVA16 (N = 6,120)	3.138	6.903	7.767	10.353	22.487	87
Other Serotypes (N = 7,135)	2.239	5.988	7.368	8.369	17.71	86
Unknown Serotypes (N = 903,332)	2.067	3.903	4.616	5.86	7.671	78

* Q_25_ and Q_75_: the first and third quartiles.

### The city-specific R0 and HIT for HFMD in Guangdong

**Fig 2:**
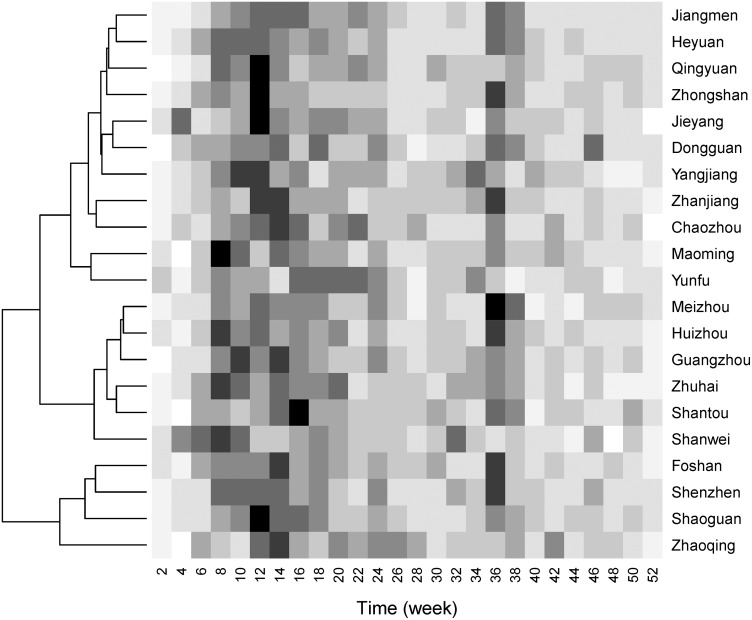
Heatmap of bi-weekly R0 by city. The dendrogram on the left side was the result of the clustering analysis.

From the heatmap of R0 (Fig 2), HFMD showed semiannual peaks of activity, including a major peak in spring and early summer (about the 12^th^ week) followed by a smaller peak in autumn (about the 36^th^ week). Based on the patterns of bi-weekly R0, 21 cities were clustered into three groups which might share similar transmission dynamics. Four of these, Zhaoqing, Shaoguan, Shenzhen and Foshan, were clustered into one group with the higher median R0 than other cities. However, these groups did not match geographically. The median R0 of each of the 21 cities ranged from 1.488 to 3.651. In terms of HIT, the proportion of each city’s population that need to be vaccinated ranged from 33% to 73%. (Table 2)

**Table 2:** City-specific R0 and HIT of HFMD in Guangdong.

City	R0			HIT (%)
	Q_25_*	Median	Q_75_*	
Zhaoqing	2.943	3.651	4.535	73
Foshan	2.749	3.650	4.326	73
Maoming	2.614	3.264	3.616	69
Shenzhen	2.441	3.111	4.432	68
Shaoguan	2.325	2.981	3.972	66
Yunfu	2.425	2.907	3.704	66
Zhanjiang	2.244	2.663	3.184	62
Qingyuan	1.947	2.561	2.757	61
Chaozhou	2.070	2.450	3.276	59
Jieyang	1.969	2.420	2.955	59
Heyuan	1.807	2.282	3.152	56
Yangjiang	1.786	2.229	2.681	55
Dongguan	1.862	2.227	2.760	55
Zhongshan	1.733	2.196	2.860	54
Jiangmen	1.893	2.165	3.264	54
Shanwei	1.531	1.958	2.525	49
Guangzhou	1.686	1.952	2.434	49
Meizhou	1.601	1.902	2.634	47
Huizhou	1.538	1.830	2.378	45
Zhuhai	1.363	1.691	2.204	41
Shantou	1.285	1.488	1.688	33

* Q_25_ and Q_75_: the first and third quartiles.

### The county-specific R0 for HFMD in Guangdong

**Fig 3:**
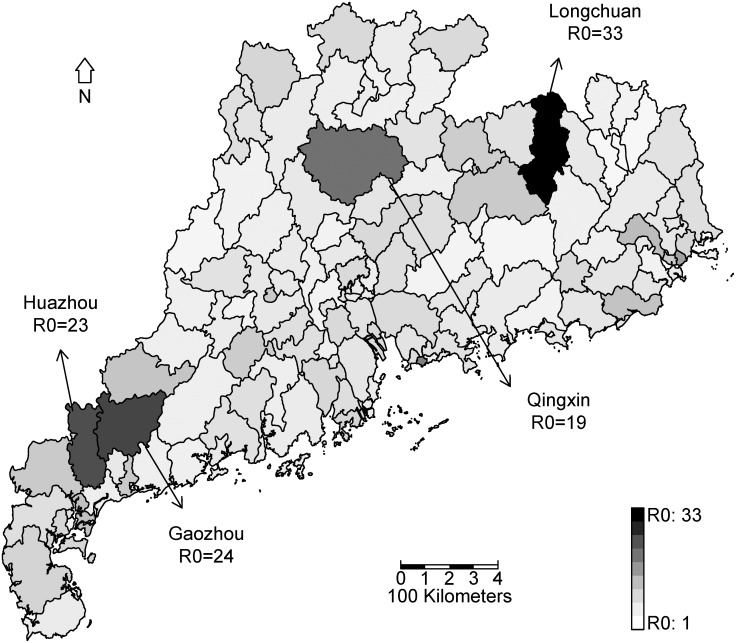
County-specific R0 of HFMD in Guangdong. This map was downloaded from OpenStreetMap (OpenStreetMap Foundation, London, United Kingdom) and processed by and R version 3.3.2 (R Core Team, Vienna, Austria).

The county-level TSIR model showed that R0 among counties varied greatly, ranging from 1 to 33 (Fig 3). Longchuan county had the highest median R0 (33). Huazhou and Gaozhou counties had the second median R0 (23 and 24). A high median R0 (19) also was found in Qingxin county. All these counties are located in the Eastern Region, Western Region, and Mountainous Region.

## Discussion

In this paper, we have estimated two principal epidemiological parameters including R0 and HIT of HFMD transmission in Guangdong Province based on surveillance data from 2009 to 2012. According to our study, the HFMD in Guangdong had a median R0 of 4.621 (Table 1), the transmission potential of which was similar to that other infectious diseases including diphtheria (R0: 6–7), mumps (R0: 4–7), polio (R0: 5–7) and rubella (R0: 6–7) [5]. All of these infectious diseases are notifiable diseases in China and were included in the WHO’s expanded program on immunization (EPI), with the exception of HFMD. For HFMD cases reported from March 2009 to February 2012 in mainland China, Yang et al. estimated an R0 of 1.392, which indicated an outbreak of HFMD will occur [10]. Our results were thus apt, because the incidence of HFMD in Guangdong was almost three times the national average [1, 24] and we updated the data to December 2012 including the increasing disease burden. Thus, to control the spread of HFMD in Guangdong, strategies such as the implementation of EPI vaccination programs should be applied. Our efforts are targeted at increasing the herd immunity to the HIT of 78% (Table 1).

For city-specific results, two peaks of R0 were found which corresponded to the observed time series of HFMD, following the Xing et al. findings published in The Lancet’s *Infectious Diseases* journal [15]. Based on the patterns for bi-weekly R0, cities were divided into three groups by clustering analysis (Fig 2). In this way, the HFMD in 21 cities can be prevented and controlled by group and more attention can be paid to the two upcoming peaks. For county-specific results, we clarified the spatial distribution of the R0 for HFMD in Guangdong (Fig 3). The counties with higher R0 were in the Eastern Region, Western Region, and Mountainous Region, all of which have lower socio-economic profiles than that of the Pearl River Delta Region. Appropriate and enhanced prevention strategies should be implemented in these counties due to the higher R0 indicating the higher potential of outbreak.

This study stood out from previous studies by its strengths. First, the TSIR model used in this paper had an acceptable goodness of fit due to bringing the historical time series into the model. The indicators for goodness of fit, including the standardized difference (0.009), the MAPE (40.94%) and Pearson’s product-moment coefficient (0.858), were all acceptable; other models, especially the simple SIR, might not render such a high correlation [8]. Second, by exploring the spatial pattern of R0 across the province and indicating the spatial variance from province to county level. Resources can be targeted effectively by the spatial patterns, an improvement over previous studies which usually focused on the temporal perspective [25]. Moreover, our study’s HIT calculations for province cities can provide the evidence to support the implementation of the country’s urgently needed vaccination program (i.e. EV71 vaccination) [14]; to the best knowledge, previous studies do not put forward this important parameter for disease control.

However, there are also some limitations to this study. The absence of age structure in the TSIR model did not allow us to assess the degree of age-focusing of vaccination. Subsequent models could be further refined to allow for transmission by age. Fortunately, the majority (95%) of HFMD cases comprised children under the age of five and our results reflect on this susceptible population. In addition, the current time series were not updated to the latest year (2015, before the EV71 vaccination was available). Since the cases of HFMD increased annually, we might have slightly underestimated its transmission. However, the quality of the fit suggested that our appeal of strong control and prevention is reasonable as a first step.

## Conclusions

Using a relatively simple mathematical model (TSIR), we detected a robust R0 and a signature of herd immunity, driving the outbreak dynamics of HFMD. Our result indicated that the epidemic of HFMD in Guangdong continues to pose a serious threat as all the R0 from models of different geographical levels were greater than one. The R0 of CVA16 cases was higher than that of EV71 cases. Counties with higher R0 including Longchuan, Gaozhou, Huazhou, and Qingxin should be paid more attention to. Targeted strategies for prevention and control such as those of WHO’s expanded programs should be implemented. An HIT of 78% might achieve an elimination of HFMD in Guangdong.

## Supporting information

S1 Dataset HFMD cases and demographic data of Guangdong, China. Monthly case counts of HFMD for Guangdong, from January 2009 to December 2012. Annual population (× 10,000) and birth counts (× 10,000) of Guangdong, from 2009 to 2012.
(XLSX)

S1 Table The estimated values of α and β obtained from each model. (XLSX)

## References

[1] National Health and Family Planning Commission of China. The National Statutory Epidemic Situation of Infectious Diseases of China. 2017-02-03 [cited 2017-03-01]. http://www.nhfpc.gov.cn/jkj/s2907/new_list.shtml.

[2] Zhu Q, Hao Y, Ma J, Yu S, Wang Y. Surveillance of Hand, Foot, and Mouth Disease in Mainland China (2008–2009). BIOMED ENVIRON SCI. 2011;24: 349–56. doi: 10.3967/0895-3988.2011.04.005 22108323 10.3967/0895-3988.2011.04.005

[3] Deng T, Huang Y, Yu S, Gu J, Huang C, Xiao G, et al Spatial-temporal Clusters and Risk Factors of Hand, Foot, and Mouth Disease at The District level in Guangdong Province, China. PLOS ONE. 2013;8: e56943 doi: 10.1371/journal.pone.0056943 23437278 10.1371/journal.pone.0056943 PMC3578924

[4] Zhang W, Du Z, Zhang D, Yu S, Hao Y. Boosted regression tree model-based assessment of the impacts of meteorological drivers of hand, foot and mouth disease in Guangdong, China. SCI TOTAL ENVIRON. 2016;553: 366–71. doi: 10.1016/j.scitotenv.2016.02.023 26930310 10.1016/j.scitotenv.2016.02.023

[5] Michigan Center For Public Health Preparedness. Basic Reproductive Rate (Ro). 2010 [cited 2017-03-01]. http://practice.sph.umich.edu/micphp/epicentral/basic_reproduc_rate.php.

[6] Public Health Action Support Team (PHAST). Epidemic theory. 2008-08-10 [cited 2017-03-01]. https://www.healthknowledge.org.uk/e-learning/epidemiology/specialists/epidemic-theory.

[7] Anderson RM, May RM. Infectious Diseases of Humans: Dynamics and Control. Oxford: Oxford University Press; 1991.

[8] F. CST, J. L. A Simple Deterministic Model for the Spread of Hand, Foot and Mouth Disease (HFMD) in Sarawak. Presented at: Second Asia International Conference on Modelling and Simulation (AMS), 2008:947–52.

[9] Roy N, Haider N. Compartmental modeling of hand, foot and mouth infectious disease (HFMD). Research Journal of Applied Sciences. 2010;5: 177–82.

[10] Yang J, Chen Y, Zhang F. Stability analysis and optimal control of a hand-foot-mouth disease (HFMD) model. Journal of Applied Mathematics and Computing. 2013;41: 99–117.

[11] Finkenstadt BF, Grenfell BT. Time Series Modelling of Childhood Diseases: A Dynamical Systems Approach. Journal of the Royal Statistical Society. Series C (Applied Statistics). 2000;49: 187–205.

[12] Finkenstädt BF, Bjørnstad ON, Grenfell BT. A stochastic model for extinction and recurrence of epidemics: estimation and inference for measles outbreaks. BIOSTATISTICS. 2002;3: 493–510. doi: 10.1093/biostatistics/3.4.493 12933594 10.1093/biostatistics/3.4.493

[13] Du Z, Zhang W, Zhang D, Yu S, Hao Y. The threshold effects of meteorological factors on Hand, foot, and mouth disease (HFMD) in China, 2011. Sci Rep. 2016;6: 36351 doi: 10.1038/srep36351 27848955 10.1038/srep36351 PMC5111081

[14] Sanicas M. Severe Hand, Foot, and Mouth Disease (HFMD) is now vaccine-preventable. 2016-08-01 [cited 2017-03-01]. http://www.impatientoptimists.org/Posts/2016/01/Severe-Hand-Foot-and-Mouth-Disease-HFMD-is-now-vaccinepreventable-at-least-in-China.

[15] Xing W, Liao Q, Viboud C, Zhang J, Sun J, Wu JT, et al Hand, foot, and mouth disease in China, 2008–12: an epidemiological study. LANCET INFECT DIS. 2014;14: 308–18. doi: 10.1016/S1473-3099(13)70342-6 24485991 10.1016/S1473-3099(13)70342-6 PMC4035015

[16] National Population and Health Science Data Sharing Platform. The Data-center of China Public Health Science. 2001-04-02 [cited 2017-03-01]. http://www.phsciencedata.cn/Share/en/index.jsp.

[17] WHO. A Guide to Clinical Management and Public Health Response for Hand, Foot and Mouth Disease (HFMD). 2011 [cited 2017-03-01]. http://www.wpro.who.int/emerging_diseases/documents/HFMDGuidance/en/.

[18] Bj X, Rnstad ON, Finkenst X, Dt BX, Rbel F, Grenfell BT. Dynamics of Measles Epidemics: Estimating Scaling of Transmission Rates Using a Time Series SIR Model. ECOL MONOGR. 2002;72: 169–84.

[19] KJ R. Modern Epidemiology. Baltimore, United States: Lippincott Williams & Wilkins; 1998.

[20] Li M, Jin Z, Sun G, Zhang J. Modeling direct and indirect disease transmission using multi-group model. J MATH ANAL APPL. 2017;446: 1292–309.

[21] Li M, Sun G, Wu Y, Zhang J, Jin Z. Transmission dynamics of a multi-group brucellosis model with mixed cross infection in public farm. APPL MATH COMPUT. 2014;237: 582–94.

[22] Wu Y, Li M, Sun G. Asymptotic analysis of schistosomiasis persistence in models with general functions. Journal of the Franklin Institute. 2016;353: 4772–84.

[23] Daubechies I, DeVore R, Fornasier M, Güntürk CS. Iteratively reweighted least squares minimization for sparse recovery. COMMUN PUR APPL MATH. 2010;63: 1–38.

[24] Guangdong Health Department. The National Statutory Epidemic Situation of Infectious Diseases of Guangdong Province, China. 2016-02-15 [cited 2017-03-01]. http://www.gdwst.gov.cn/a/yiqingxx/.

[25] Zhu Y, Xu B, Lian X, Lin W, Zhou Z, Wang W. A Hand-Foot-and-Mouth Disease Model with Periodic Transmission Rate in Wenzhou, China. ABSTR APPL ANAL. 2014;2014: 234509.

